# Reconstructive surgery of difficult urethrocutaneous fistula following gender‐affirming surgery

**DOI:** 10.1002/iju5.12762

**Published:** 2024-08-27

**Authors:** Kazuna Matsuo, Miki Kanbe, Fumitoshi Sakamoto, Yuzuru Kamei, Shusuke Akamatsu

**Affiliations:** ^1^ Department of Urology Nagoya University Graduate School of Medicine Nagoya Aichi Japan; ^2^ Department of Plastic and Reconstructive Surgery Nagoya University Graduate School of Medicine Nagoya Aichi Japan; ^3^ Urology Chubu Rosai Hospital Nagoya Aichi Japan

**Keywords:** gender‐affirming surgery, pedicle flap, reconstructive surgery, transgender man, urethrocutaneous fistula

## Abstract

**Introduction:**

In Japan, transgender individuals have historically had limited therapeutic options, prompting many to seek gender‐affirming surgeries in private or foreign clinics because of restricted access to public hospitals. This has led to challenges for patients undergoing surgery.

**Case presentation:**

A transgender man underwent surgery at a private clinic and experienced recurrent complications. Subsequent examination at another clinic and our hospital revealed limited medical records, complicating our understanding of this case. After a detailed investigation, the urethrocutaneous and urethrovaginal fistulas were identified and addressed by joint urologists and plastic surgeons, resulting in no recurrence after 1 year.

**Conclusion:**

This case underscores the importance of thorough preoperative assessment with a flexible mindset, emphasizing the need to avoid being misled by inadequate records or appearances in complication management of gender‐affirming surgery. Collaborative efforts among healthcare professionals based on comprehensive evaluations lead to safer complication treatments.

**Abbreviations & Acronyms**
ALT: anterior lateral thigh
GAS: gender‐affirming surgery

**Keynote message**

This case emphasizes the complexity of a fistula following gender‐affirming surgery. This highlights the significance of meticulous surgical planning and the effective management of complications. Collaboration among healthcare professionals is pivotal for advancing gender‐affirming care and ensuring the best possible patient outcomes.

## Introduction

In Japan, gender‐affirming surgery (GAS) is performed in accordance with the guidelines of the Japanese Society of Psychiatry and Neurology. In principle, at least one of the primary surgeons or a group of primary surgeons must be certified by the Japanese Society of Gender Identity Disorder. Historically, however, therapy for transgender individuals in Japan has been challenging, with many patients not undergoing GAS in public hospitals. Consequently, some patients seek therapy at private plastic clinics or foreign clinics/hospitals, often at their own expense and without insurance coverage.^1^ Although public hospitals have begun to provide care to these patients, their numbers remain limited.

## Case presentation

The patient was a 46‐year‐old transgender man who underwent GAS at a private plastic clinic. He explained that he had experienced a recurrence of a urethrocutaneous fistula after three surgeries at the clinic. Consequently, our hospital became involved in patient care.

**Fig. 1:**
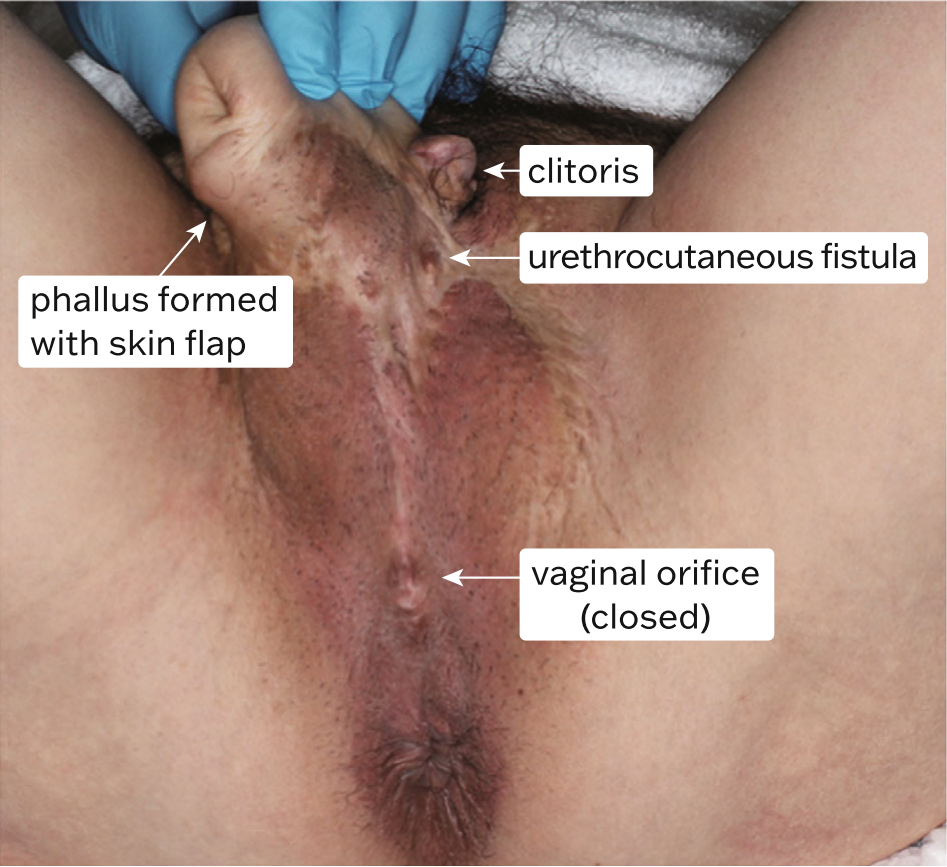
Pubic appearance at visit.

Patient had a surgical description and consent form from the clinic primarily consisted of GAS plans, providing limited information about the urethra. The preoperative plans indicated urethral lengthening with a vaginal vestibular flap and phalloplasty with an anterior lateral thigh (ALT) flap. External examination revealed a well‐developed phallus and a closed vagina (Fig. 1). Scrotal tissue formed by the labia majora had been used for fistula repair.

### Examination

Upon examination, a urethrocutaneous fistula was observed in the plastic phallus. Cystoscopy confirmed urethral stricture. Further investigation of the urethra revealed a fistula leading to a dilated native vagina that was incompletely removed during GAS. The native vagina was covered with a flap and was not visible. Evaluating the urethra and bladder in a retrograde direction was impossible because the cystoscope moved forward in the orientation of the urethrovaginal fistula.

### Operation

**Fig. 2:**
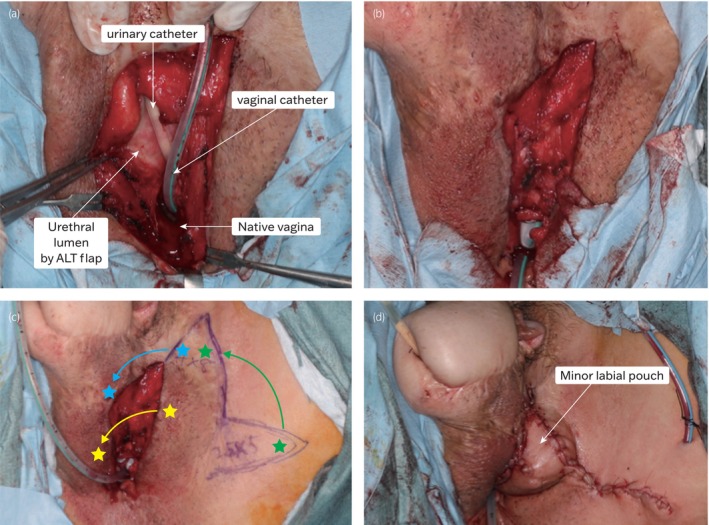
(a) Removed urethrocutaneous and urethrovaginal fistulas, vertically incised ALT flap. Inserted Foley catheter intraoperatively as a marker in the naive vagina. (b) Closed urethra, covered with surrounding tissue. (c) Rotated a bilobed flap to patch the urethral closure site, in addition, the flap was placed over a partial circumferential vaginal orifice, further rotated second flap from inner thigh to relieve tension at the bilobed flap donor site. Carefully positioned flap to maintain blood flow. (d) After fistula closure, intentionally preserved untrimmed dog‐ear at the site of harvested inner thigh skin flap, resembling a minor labial pouch.

A suprapubic cystostomy was created and a urethral catheter was placed under fluoroscopic guidance. Subsequently, a urethrocutaneous fistula was identified and a guidewire was passed through. The guidewire was used as a marker to make an incision and reach the plastic urethra. This incision was made toward the central side to reach the vaginal cavity, and a urethrovaginal fistula was identified. A Merkmal catheter was placed in the vagina, and both fistulas were excised en bloc (Fig. 2a). There was sufficient residual tissue to ensure an adequate urethral lumen. The neourethra was covered using a double‐layered suture technique, wherein the fascia was sutured over the sutured neourethra along the ventral longitudinal axis using absorbable sutures (Fig. 2b), and a bilobed flap containing the perforating branch of the internal labial artery was raised from the labia majora and the inner thigh to shift the suture line (Fig. 2c). A dogear was formed upon suturing the edge of the flap around the vagina and urethral defect. The dogear is usually removed and flattened; however, in this case, it was left as a small scrotum (Fig. 2d), and the original vaginal orifice was left open to facilitate the drainage of any fluid. Two drains were placed at the skin flap site.

**Fig. 3:**
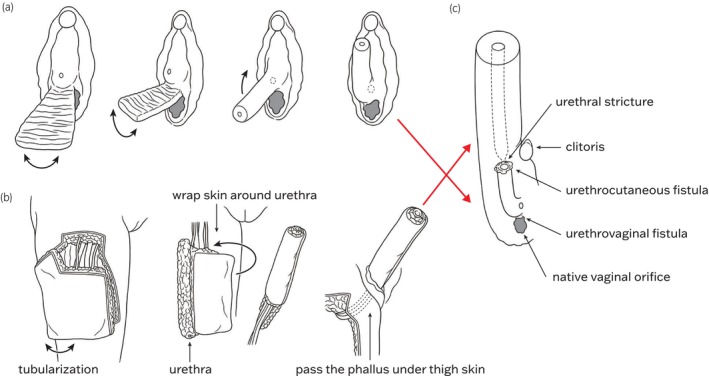
(a) Tubular formation of the anterior vaginal wall flap, performing urethral lengthening procedure. (b) The thigh skin is tubularized to form urethra and wrap skin around urethra (role‐in‐role procedure) for phallus construction. Pass the phallus under thigh skin. (c) Urethral extension within the phallus from (b), anastomosed to the lengthened urethra with the anterior vaginal wall flap covering the vaginal orifice (estimated). Subsequently, indicating the sites where urethrocutaneous fistula and urethrovaginal fistula were formed.

Consequently, the following process was considered to have occurred for the GAS: First, the patient's GAS included removal of the uterus and ovaries and urethral lengthening using the anterior vaginal wall flap (Fig. 3a). Second, neophallus construction using the ALT flap (Fig. 3b), urethral reconstruction using the ALT flap and anterior vaginal wall flap (Fig. 3c), and closure of the vaginal orifice using a portion of the ALT flap. A urethrocutaneous fistula was precisely located at the junction of the neourethra, and a pinpoint urethral stricture was present distal to the fistula and covered by a portion of the ALT flap. Additionally, a urethrovaginal fistula had formed proximally (Fig. 3c).

**Fig. 4:**
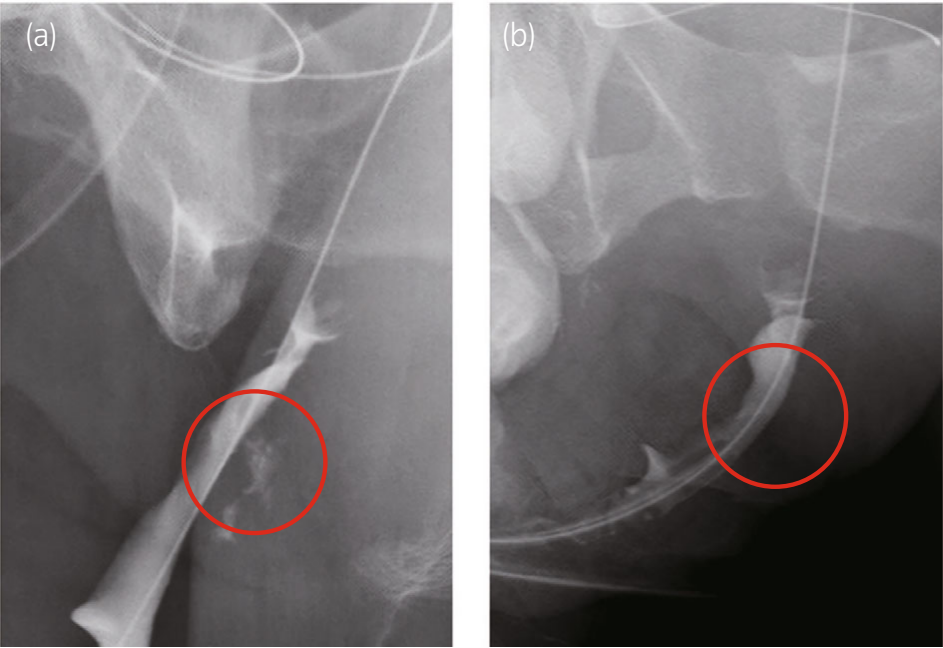
(a) Postoperative urethrogram at 2 weeks. Detected minor leak, no contrast extravasation into the skin. (b) Urethrogram postgauze compression of the leaking area (4 weeks postoperative). No contrast leakage from the urethra.

Postoperatively, when the drainage output from both sides remained below 5 mL, the drains were removed. On postoperative day 13, a urethrogram revealed closure of the urethrovaginal fistula, but a small leak from the urethrocutaneous fistula closure site (Fig. 4a). Minor leakages were managed conservatively with gauze compression. One month postoperatively, the leakage had resolved (Fig. 4b).

## Discussion

Urethral lengthening in GAS commonly utilizes donor flaps, such as radial forearm or ALT flaps. A previous study summarized the complications associated with these procedures, including wound complications, stricture, fistula, and flap stenosis with ALT flap use.^2^ Several mechanisms underlie urethral fistula formation.^3^, ^4^ One potential mechanism involves pressure from the urinary flow, which tends to follow the path of least resistance. Resistance near the ventral side of the urethra can disrupt the nonventral suture line, leading to fistula formation. Moreover, in GAS, *the anterior vaginal wall flap is tubularized surrounding the native urethral meatus*, altering the position and orientation of the urethral opening, and lengthening it. Due to the high elasticity of the vaginal tissue, it can expand excessively, creating a bag‐like structure and placing tension on the suture line. This can lead to tissue thinning and vascular compromise. Other factors contributing to urethral fistula formation include urethral, vaginal, and external genital atrophy due to testosterone therapy and increased intraurethral pressure secondary to urethral stricture. The latter leads to pressure elevation during urination, especially in the presence of a bottle‐necked obturator in the urethra. This causes an increase in pressure during urination ahead of the distal portion of the neck, potentially destroying the relatively weak surrounding tissues.

Preventive measures include thickening the ventral tissue of the neourethra (using full‐thickness grafts, fasciocutaneous flaps, and role‐in‐role techniques), avoiding suture lines in areas subject to urinary pressure, and utilizing well‐vascularized anterior vaginal wall flaps during urethral lengthening procedures.^3^, ^4^

In the present case, the complexity of the procedure and the limited information from a private plastic clinic posed significant challenges. Initially, only a urethrocutaneous fistula was suspected. However, covering the vaginal cavity resulted in a urethrovaginal fistula, necessitating extensive incision due to the presence of two fistulas. Fortunately, the wide enough lumen facilitated closure without issue. The decision not to reconstruct the vaginal canal aimed to preserve the cavity, providing a drainage route and preventing fistula reformation due to reinfection. This case highlights the importance of cautious vaginal cavity closure.

## Conclusion

This case underscores the challenges of gender‐affirming surgery and complication management, emphasizing the importance of comprehensive knowledge and a flexible approach to avoid being misled by appearances or insufficient descriptions. Sharing experiences and knowledge can contribute to ongoing improvements in gender affirmation practices, ultimately providing better care for transgender and gender‐diverse individuals on their journey toward gender affirmation.

## Author contributions

Kazuna Matsuo: Writing – original draft. Miki Kanbe: Writing – review and editing. Fumitoshi Sakamoto: Writing – review and editing. Yuzuru Kamei: Supervision. Shusuke Akamatsu: Conceptualization; supervision; writing – review and editing.

## Conflict of interest

The authors declare no conflict of interest.

## Approval of the research protocol by an Institutional Reviewer Board

Not applicable.

## Informed consent

Written informed consent was obtained.

## Registry and the Registration No. of the study/trial

Not applicable.
